# Editorial Practices of African Journals: A Qualitative Analysis from Kenya, Ethiopia, Nigeria, and Mozambique

**DOI:** 10.12688/gatesopenres.16376.1

**Published:** 2026-02-04

**Authors:** Patrick Amboka, Daniel Krugman, Abel Simiyu, H Kariuki, Benard Ondiek, Nosa Orobaton, Emmy Igonya, Alphonsus Neba, Marta Vicente-Crespo, Julius Kirimi Sindi

**Affiliations:** 1 African Population and Health Research Center, Nairobi, Nairobi County, Kenya; 2 Brown University, Providence, Rhode Island, USA; 3 Bill & Melinda Gates Foundation, Seattle, Washington, USA; 4 University of the Witwatersrand, Johannesburg, Gauteng, South Africa

**Keywords:** LMIC, Journal visibility, Discoverability and indexing of African journals, Existing capacity, Editorial practices, Journals trustworthiness, Underlying factors, Journal credibility

## Abstract

**Introduction:**

Information on journal visibility helps researchers decide where to publish. Some quality indicators used are directly associated with the journal’s editorial practices. By understanding the barriers, challenges, and opportunities, this study aims to explore existing editorial practices among African journals, explore the underlying factors affecting the editorial practices of African journals, and understand the views and preferences of authors regarding the choice of journals for publication.

**Methodology:**

This study triangulated the sources of information and qualitative design data-gathering techniques to allow for nuances and deeper insights into the performance and visibility of African Journals. We conducted In-depth Interviews (IDIs), Key Informant Interviews (KIIs), and Focus Group Discussions (FGDs) in Kenya, Ethiopia, Nigeria and Mozambique. The study population comprised journal editors-in-chief, representatives from African-wide journal databases/indexers, institutional repository representatives, and authors. A purposive sampling technique was used to identify participants. Ethical approval was obtained from the relevant bodies. Qualitative data from the audio-recorded interviews were transcribed using MS Word and exported to NVivo software for analysis.

**Results:**

The key structural issues on editorial practices among African journals established by the study included adherence to internationally accepted editorial practices on peer review decision-making and challenges in implementing measures of transparency and rigor. Some of the underlying factors affecting African journal editorial practices that were highlighted included financial constraints, challenges in peer review, challenges in maintaining editorial integrity, and challenges in technological and digital infrastructure. African journals also face challenges of credibility and trustworthiness among authors. Participants outlined how the longstanding neglect of African journals and lack of funding have created cultures of editorial mismanagement, publishing inconsistency, and other logistical issues, all of which contribute to perceptions of African journals as inferior to Northern ones.

## Introduction

In recent years, research has focused on the distribution of power. Led by scholars in and from the global South, “unfair knowledge practices” that allow global North actors and institutions to dominate knowledge production, and thereby, displace and erase that which is produced in the South, have increasingly been named, mapped, and understood. ^1^ Through the frameworks of “epistemic injustice,” how these practices are structurally engrained into the field as much as it is facilitated by individual actions is well-established. ^2^ From the underrepresentation of marginalized voices and ways of knowing in dominant platforms to the systematic exclusion of peripheral cultural and technical expertise in the field, ^1^ dominance by a small number of privileged experts is the norm of the field—one that is baked into its financial, social, and political structuring. ^3^ The value of African journals lies in their potential to decolonize knowledge production by providing a platform for contextually relevant research that addresses African priorities and realities. They serve as critical spaces for amplifying African scholarship, fostering regional collaboration, and building local research capacity. Despite structural challenges, strengthening African journals can contribute to more equitable global scientific discourse and ensure that African voices shape research that impacts the continent.

The visibility of journals refers to how easily a journal and its published articles can be found, accessed, and cited. It typically includes indexing in major databases, open access, impact factor, and metrics, website, search engine optimization, promotion and dissemination, and inclusion in library catalogs and reporting. African journals play a pivotal role in shaping the continent’s academic landscape, and can contribute significantly to the global dissemination of knowledge. African journals are invaluable platforms for scholars and researchers to share their findings, ideas, and innovations, while amplifying voices that are often underrepresented in mainstream international publications.

Metrics such as Impact Factor (IF), CiteScore, and SCImago Journal Rank (SJR) dominate discussions on journal visibility, guiding the decisions of researchers and policymakers alike. ^4, 5^ Despite the global reliance on these metrics, African journals remain underrepresented on major indexing platforms and are poorly cited globally. African researchers frequently publish their work in international journals rather than African-based journals, compounding the invisibility of African scholarship. ^6^ This disparity marginalizes the Global South in global knowledge production, where only 31 countries account for 97.5% of the world’s most-cited articles. ^7^

Journal visibility is essential for achieving global recognition and impact, as determined by inclusion in indexing platforms such as Web of Science, Scopus, and Google Scholar. ^8^ Metrics such as Impact Factor (Clarivate) and CiteScore (Elsevier) are widely used to rank journals based on their citation rates. ^6^ However, these metrics often reflect systemic biases favoring Global North journals because of differences in resource allocation and infrastructural support. ^9^ Both the International Network for Advancing Science and Policy (INASP) and the Directory of Open Access Journals (DOAJ) work to enhance the visibility and accessibility of quality research in developing regions including Africa. INASP assesses and indexes journals based on publishing practices and standards through its Journal Publishing Practices and Standards (JPPS) framework, focusing on supporting journals in developing countries. ^10^ The DOAJ indexes open-access journals that meet strict criteria for quality and transparency, emphasizing global accessibility to research outputs. ^11^ Recent studies emphasize the advantages of Open Access (OA) publishing for increasing citations, with OA journals reporting an 18% citation boost compared with non-OA journals. ^12^ Platforms such as DOAJ offer quality assurance for OA journals, making them more visible globally. However, many African journals struggle to meet the stringent quality standards required for inclusion in such platforms. ^13^

The Committee on Publication Ethics (COPE) supports and educates publishers and editors by providing resources on publication ethics, which makes journals attract the best manuscripts. ^14^ A journal’s membership in COPE conveys to authors, reviewers, and readers that it adheres to the recommended core practices for the highest ethical standards in research publishing. ^14^ Editorial practices are central to journal visibility. Key issues include limited access to plagiarism detection tools, lack of skilled editorial staff, and inadequate peer review processes. ^15^ Studies have shown that the absence of robust editorial workflows and transparency negatively impacts journal credibility and visibility. ^16^ Financial challenges have further compounded these issues. African journals often lack funding to maintain editorial independence, pay editorial staff, or invest in digital infrastructure. ^17^ The reliance on volunteer editors, who juggle academic duties alongside journal responsibilities, contributes to delays and inconsistencies in the publication processes. ^18^ These systemic barriers are further exacerbated by biases in editorial standards and perceptions of African research as less prestigious, making the need for localized solutions more urgent than ever.

The global research ecosystem is characterized by perceptions that African journals are of lower quality, further marginalizing them in scholarly discourse. ^19^ This bias is rooted in the historical legacies of colonialism, which continue to influence the valuation of research outputs from the Global South. ^20^ In addition, African journals are often excluded from high-impact platforms, perpetuating a cycle of invisibility and lack of recognition. ^21^ Despite these challenges, several initiatives have shown promise in this regard. Platforms such as African Journals Online (AJOL) have improved the visibility of indexed African journals, demonstrating the potential for regional efforts. ^22^ The persistent invisibility of African journals is a critical issue hindering the dissemination of valuable research findings, limiting the global recognition of African scholarship, and perpetuating inequities in knowledge production. Although existing metrics and platforms provide tools for assessing visibility, they fail to address the structural and contextual challenges unique to African journals. Editorial practices, ranging from peer review to digital integration, are particularly underexplored despite their central role in determining visibility.

The visibility of African journals is a critical issue that must be addressed. Despite the significant contributions of African researchers and scholars, there is a persistent lack of global recognition and visibility for journals published in Africa. This lack of recognition and visibility not only hampers the dissemination of valuable research findings, but also hinders the development and progress of African academia as well as decolonizing education. By understanding the barriers, challenges, and opportunities, this paper aimed to explore existing editorial practices among African journals, explore the underlying factors affecting the editorial practices of African journals, and understand the views and preferences of authors regarding the choice of journals for publication.

## Methodology

### Reporting standards

This study adhered to the Enhancing the Quality and Transparency of Health Research (EQUATOR) reporting guidelines, specifically applying the Consolidated Criteria for Reporting Qualitative Studies (COREQ) framework. ^23^ The use of the COREQ ensures that this study meets the highest standards for transparency, rigor, and reproducibility in qualitative research.

### Study design

This qualitative study sought to establish existing editorial practices and the underlying factors affecting the editorial practices of African journals, and to understand the preferences of authors regarding the choice of journals for publication by exploring editorial practices among African journal editors. In this study, we triangulated the sources of information and qualitative design data-gathering techniques to allow for nuances and deeper insights into the performance and visibility of African Journals. In-depth Interviews (IDIs) and Key Informant Interviews (KIIs) were conducted with stakeholders, including journal editors-in-chief, representatives from African-wide journal databases and indexers, institutional repositories, institutional heads of research, and authors or potential publishers. The first set of Focus Group Discussions (FGDs) involved journal editors-in-chief to explore the broad spectrum of challenges and opportunities that influence African journal visibility. The second set of FGDs was conducted with the authors to understand their motivations, preferences, and decision-making processes when selecting journals for publication. The combination of IDIs, KIIs, and FGDs ensured a comprehensive understanding of the study topic from multiple perspectives. The FGDs were done after the key informant interviews and in-depth interviews to get clarity on aspects that were otherwise not clear during the interviews.

### Stakeholder engagement

To ensure the success of the study, initial meetings were held with stakeholders to discuss the data collection plan and secure their buy-in. An inception meeting was convened with investigators from the African Population and Health Research Center (APHRC) and country-specific stakeholders to establish project goals, timelines, and criteria for participant selection. During this meeting, stakeholders played a pivotal role in identifying the key focal persons who would facilitate engagement and communication with potential participants. This approach ensured that the recruitment process was inclusive and representative of the diverse perspectives necessary for this study. To foster transparency and alignment, official communication outlining the study’s objectives, methodologies, and timelines was disseminated to relevant institutions through designated focal persons. This structured approach helped to secure institutional support and participant engagement throughout the study.

### Sampling strategy

An initial search of Kenyan journals identified 120 journals hosted by universities and research institutions. We also found 107 journals in Ethiopia hosted in Ethiopian Journals Online, and 240 journals hosted by Nigerian institutions of higher learning. This served as a reference point for generating an adequate sample size. The study utilized a purposive sampling technique and targeted African journal editors-in-chief, African-wide journal databases/indexers (representatives of the four countries), institutional repositories, journal editors-in-chief, authors, and institutional heads of research from the four countries. Purposive sampling was utilized to ensure equal representation of the study participants based on thematic areas (humanities, social sciences, health sciences, etc.). This selection ensured that the study captured a holistic view of the challenges and opportunities affecting journal visibility, drawing on perspectives on publication, indexing, institutional oversight, and authorship. Table 1 summarizes the categories of participants included in the study.

**Table 1.** Summary of participants included in the study.

Participant category	Type/Number of interviews		Country
	IDI	FGD	
Journal editors-in-chief	30	1	Kenya
Authors (Not journal editors-in-chief )	30	1	Kenya
Institutional repositories representatives	20	-	Kenya
Institutional heads/Research directors)	10	-	Kenya
African-wide repositories/indexers	2	-	African-wide
Journal editors-in-chief	-	1	Nigeria
Authors (Not journal editors-in-chief )	-	1	Nigeria
Journal editors-in-chief	-	1	Ethiopia
Authors (Not journal editors-in-chief )	-	1	Ethiopia
Authors (Not journal editors-in-chief )	-	1	Mozambique
**Total**	**92**	**7**	

### Study location

The study was conducted at universities and research institutions across Kenya, Ethiopia, Nigeria, and Mozambique. The choice of these countries reflects their varying research outputs, infrastructural capacities, and representations within the broader African scholarly ecosystem. By focusing on these nations, this study aimed to provide a comprehensive and representative understanding of journal editorial practices, the underlying factors of editorial practices, and authors’ perspectives on African journal credibility and trustworthiness in different contexts.

### Inclusion criteria

Participants were selected based on the following criteria:

1. **Journal editors-in-chief** from Kenya, Ethiopia, Nigeria, and Mozambique with experience in managing African journals.
2. **Representatives of institutional repositories** in the four focus countries.
3. **Representatives from African-wide journal databases/indexers** with operations in the study countries.
4. **Authors** with prior or intended publication experience in African journals.
5. **Institutional heads of research** were responsible for overseeing research dissemination at their respective institutions.

### Exclusion criteria

1. Authors who were non-scholars (Not publishing in scholarly journals)
2. Editor-in-chief from journals focusing on Africa but not hosted in African institutions

### Data collection

**Training of research assistants (RAs)**

The RAs were trained in moderating, listening, and tape recording without displaying any judgmental attitudes towards the participants or the information being received. This study was conducted in accordance with the ethical standards outlined in the Declaration of Helsinki. The training included focused sessions and exercises regarding the meaning and process of informed consent (see more details in the Ethical Considerations section), the importance of protecting the privacy of subjects, and the confidentiality of information obtained from them.

**Pretesting and refinement of tools**

The study tools, including interview and discussion guides, underwent a pre-testing process with a small group of participants whose characteristics mirrored those of the study population. This step ensured the clarity, cultural relevance, and reliability of the tools. The pretesting process identified potential challenges, allowing the research team to make necessary adjustments before full-scale data collection. The pretest also identified some inconsistencies in the tools and helped identify ambiguous and unclear questions. The tools were therefore adjusted accordingly. This step was instrumental in minimizing bias and enhancing the overall quality of the data collected. Pre-testing interviews and discussion guides ensured that the tools were culturally and contextually appropriate, enhancing clarity and minimizing potential biases. This step improved the relevance and reliability of the collected data, contributing to the overall quality of the study.

**KIIs and IDIs**

The study employed KIIs and IDIs to collect data in Kenya, and FGDs to gather detailed, context-rich information on African journal editorial practices and the underlying factors that affect them, as well as the decision-making process of the potential authors in Kenya, Nigeria, Ethiopia, and Mozambique. The KIIs are conducted with individuals who have expert knowledge or hold influential positions, such as policymakers or organizational leaders, to gain strategic insights on broader issues. In contrast, IDIs involve individuals with personal experience of the subject matter, allowing researchers to explore detailed narratives, perceptions, and lived experiences. While KIIs focus on systems and institutional perspectives, IDIs provide a more individual and nuanced understanding of a topic. Participants included journal editors-in-chief, representatives from institutional repositories, representatives from African-wide journal databases and indexers, and authors.

These methods were chosen to provide comprehensive insights into editorial practices and the underlying factors to editorial practices. IDIs with authors are specifically designed to explore their preferences and motivations when selecting publication venues, offering valuable perspectives on how these decisions impact the visibility of African journals.

The KIIs and IDIs were conducted exclusively in Kenya because of data saturation observed during FGDs in Ethiopia, Nigeria, and Mozambique. The FGDs revealed consistent themes across countries, allowing the study to concentrate its detailed interviews in Kenya without sacrificing data diversity or depth. This approach ensured efficient resource use, while maintaining the reliability and richness of the findings. The interviews were conducted only with the interviewer and the notetaker. The guides were not provided to the respondents before the interview, and all interviews were researcher-administered. There was one repeat interview conducted at the request of the respondent. The respondent opted to repeat the interview to provide comprehensive insight into the visibility of African journals. We invited 35 editors-in-chief to participate in the study and 30 accepted the invitation. However, in other categories, all invited participants accepted the invitation to participate in the study. The interviews were recorded and the audio was verified by a team lead before approval for transcription and data entry.

**FGDs**

FGDs were conducted in Kenya, Ethiopia, Nigeria, and Mozambique. FGDs explored shared and divergent viewpoints across regions. In Kenya, FGDs followed KIIs and IDIs, delving deeper into emerging themes, whereas in Ethiopia, Nigeria, and Mozambique, FGDs alone achieved data saturation. Two FGDs were conducted in each of these countries: one with journal editors-in-chief and the other with authors. Each group consisted of twelve purposively selected participants, ensuring a wide range of perspectives. Trained qualitative researchers moderated the discussions using structured guides informed by preliminary findings from the KIIs and IDIs in Kenya. This step-by-step process allows FGDs to comprehensively explore existing themes while uncovering new insights. FGDs enriched the study by identifying commonalities and variations in participant experiences, offering a nuanced understanding of the challenges and opportunities for enhancing African journal visibility. Themes such as existing editorial practices were explored in detail, contributing significantly to the study’s findings. Because the discussion was too long, there were breaks within the sessions.

**Data saturation**

As the KIIs and IDIs were only performed in Kenya, the FGDs conducted in Ethiopia, Nigeria, and Mozambique provided similar perspectives. The consistency of the findings across countries confirmed thematic saturation, ensuring that the data collected were comprehensive and representative.

**Field notes and observations**

Field notes were taken during all KIIs, IDIs, and FGDs regarding contextual factors and emerging insights. These notes complemented the audio recordings, enriching the qualitative data and contributing to the depth of thematic analysis.

**Duration of interviews and discussions**

The duration of the interviews and discussions varied depending on the participant group and the method ^24^ ( Table 2).

**Table 2.** Average duration of interviews and discussions.

Method	Participant group	Average duration
**IDIs**	Authors	1 hour
**IDIs**	Journal editors-in-chief and institutional heads of research	2 hours
**KIIs**	Representatives from institutional repositories and African-wide databases/indexers	2 hours
**FGDs**	Authors	2 hours
**FGDs**	Journal editors-in-chief	4 hours

### Data analysis

Data collected through KIIs, IDIs, and FGDs were audio-recorded, transcribed using MS Word, and imported into **QSR NVivo 14** for analysis. ^24^ The software used can be downloaded on the Lumivero website ( https://lumivero.com/products/nvivo/). Predefined themes based on the study objectives guided the initial coding process, whereas open inductive analysis allowed for the identification of emerging themes, ensuring a comprehensive exploration of the data. The analysis process included the following steps.

1. **Coding**: Transcripts were analyzed using a combination of predefined themes based on the study objectives and open inductive content analysis to identify emergent themes. The themes were extracted from the interview guides (IDIs, KIIs, FGDs).
  ○ A coding framework was developed and two independent coders coded the transcripts. ^33^ Both the researchers and coders agreed on the coding framework. Before starting the coding process, a coding comparison query was run in NVivo and the percentage agreement between the two coders was 93%. The analysis was done per the study objectives. The coders were supervised by the researchers throughout the coding process. Queries that came up from the coding were instantly dealt with.
2. The data were organized into thematic areas. This approach identifies patterns, relationships, and divergences across participants’ experiences.

The use of NVivo software enhances the organization and depth of analysis, ensuring methodological rigor and reliability. ^24^

### Ethical considerations

The protocol and other study documents were first approved by APHRC’s Institutional Review Board (IRB) and later by the Amref Ethics and Scientific Research Committee (ESRC), Kenya (Reference number: ESRC P1677/2024). The Amref ESRC approval was granted on June 18, 2024. Members of the study team underwent ethical training for researchers and received valid certifications for the study period.

A written informed consent to participate in the study was sought from the potential participants. They were asked to sign consent forms before the start of data collection *.* Specifically, they were adequately informed about the purpose of the study and the methods used. In addition, they were informed about the institutional affiliation of the researchers, anticipated benefits and potential risks and follow-up of the study, discomfort it may entail, the right to choose to participate or not participate in the study, or to withdraw consent to participate at any time, without any reprisal whatsoever. Measures were taken to ensure confidentiality of the information they provided. Participation in this study was voluntary. The participants were informed of the length of the interview. For the physical interviews, participants were compensated for the transport they used to come to the interview venues.

The results were anonymized at all points of presentation or data sharing. Confidentiality was maintained at all times through the training of the RA on the meaning of confidentiality and mechanisms that maintain confidentiality during and after data collection. The participants’ database, with names, phone numbers, email addresses, and other relevant information, was developed. The data were stored on password-protected computers, accessible only by the members of the study team. The results were presented in reports in an aggregated manner, such that responses could not be traced back to individual participants.

## Results

### Editorial practices among African journals

This study examined several journal editorial practices and processes in Africa. The following emerging themes were reported: criteria for selecting manuscripts that will undergo peer review, adherence to editorial practices, and measures of transparency and rigor.

### Selecting manuscripts that will undergo peer review

Study participants discussed several criteria for manuscript selection as part of the editorial practices currently in place. When a journal receives a manuscript, the process typically begins with an initial submission through an online system or email, in which the article undergoes a preliminary review by the editorial office. The responsibility for receiving articles typically falls on the editorial office staff and sometimes the editor-in-chief. The editorial office staff manages the administrative aspects of manuscript submissions, ensuring that authors upload their articles correctly via the journal’s submission system or through email and that all required documents are included. Desk rejection decisions in the journals are typically made by the editor-in-chief or handling editors during the initial screening phase, before peer review. They assess whether the manuscript fits the journal’s scope, meets minimum quality standards, and follows submission guidelines. Common reasons for desk rejection include poor alignment with the journal’s focus, methodological weaknesses, lack of originality, or poor presentation or language, decisions that help manage reviewer workload and maintain the journal’s Ifstandards. Ghost authorship can raise ethical concerns about transparency and accountability, leading editors to question the integrity of a manuscript. As a result, journals may reject such submissions at the desk review stage to uphold ethical publishing standards and protect the credibility of the peer review process. If the manuscript passes this stage, the editor-in-chief assigns it to subject matter experts who will serve as peer reviewers.

Sometimes journals usually receive articles through a call for papers, which is a structured process inviting authors to submit manuscripts for special issues or on specific topics. This process often begins with an announcement outlining the theme, submission guidelines, and deadlines. In this case, the call for papers often specifies whether they will be submitted through the journal’s submission system or through email. Email submission is usually through journal’s email. First, the editorial team receives the journal via email and checks for completeness and adherence to submission guidelines before sharing it with the journal editor-in-chief. According to the participants, subject-specific is key in determining journals to submit manuscripts to. This criterion for a journal article selection ensures that submitted articles align with the journal’s focus, as explained by a journal editor-in-chief *(JE06_KII_KE)* from Kenya.

Participants indicated that language was a determining factor in manuscript selection. Journals only select manuscripts in their preferred language, such as English, French, or Spanish. Manuscript selection is determined by the journal’s designated language, and should meet the required standards of grammar, style, and academic tone. Articles that do not meet these language standards may be rejected or returned for revision to ensure that the research is presented clearly and professionally to the target audience *(IR15_KII_KE, Librarian_Kenya).*

Article publication speed was also discussed during interviews as a key factor in authors’ journal selection. Journal publication speed influences authors’ selection, as many prefer journals that provide timely processing of their research to maintain relevance. This is supported by an author *(FGD_Authors_KE)* from Kenya ‘Number two will also be the time it takes before the paper is fully published and that sometimes we look at the papers that have already been published from the time that paper was submitted to the time that paper was published.’

Challenges affecting the selection of manuscripts for review were mainly financial constraints and technological and digital infrastructure. For instance, according to the participants, the process of receiving and selecting manuscripts to undergo peer review is affected by financial resources. This study suggests that limited financial resources are required to support the operational costs of the journal, including editorial office staff salaries. Inadequate financial resources also affect the adequacy of other resources, such as human resources and time. As was reported by a journal editor-in-chief *(JE_FGD- KE)* from Kenya. ‘We are hiring staff to be the front desk and perform all the processing and the back end. There is a salary for doing so.’ According to the editor, financial constraints affect journals’ inability to hire staff to support the selection process.

Technological challenges and digital infrastructure limitations have also been reported in the selection of the manuscripts. Technological challenges and digital infrastructure can either be linked back to financial constraints or stand as independent factors affecting the selection of manuscripts that will undergo peer review. Among technological and infrastructure challenges is the lack of a proper portal for the submission of papers. Rather, manuscripts are submitted through email to the editors, complicating the process for both journals when receiving manuscripts that will undergo peer review. When receiving manuscripts through email, the editor-in-chief first reviews them to assess their quality and relevance before forwarding them to the managing editor or an editorial assistant at the front desk for administrative checks and processing. The struggle makes the journal’s processes from receiving the manuscript to publication not automatic and seamless. The technological challenges further make journals struggle with indexing, as reported by an institutional research head from Kenya.

“ *African journals are really struggling in terms of visibility and indexing. But then, that has several implications in the editorial process because when you submit a paper to an African journal, most of the time it is via email. They don’t have a proper portal where you can submit and then get that transitioned to a handling editor and then eventually into the identification of reviewers who are relevant in that particular area*” Institutional Research Head, Kenya *(IH05_KII_KE).*

Furthermore, while digital platforms are critical for journals, the availability of reliable digital platforms for handling manuscript uploads, communication, and tracking is important. Establishing and maintaining reliable and professional digital platforms can be caused by either financial constraints or a lack of expertise. A journal editor-in-chief editor from Kenya said, ‘Issues to do with technical and infrastructure limitations where you find that we face challenges establishing and maintaining a reliable and professional digital platform’ *(JE30_KII_KE).*’

### Adherence to internationally accepted editorial practices on peer review decision-making

Adherence to international editorial practices ensures fairness, transparency, and consistency in the manuscript review and publication processes. Globally recognized standards include ethical guidelines, conflicts of interest disclosures, and peer review integrity. The study participants revealed adherence to peer review practices in several journal journals in Africa. These reviewers evaluated the article for its originality, validity, findings, methodology, and overall contribution to the field. The reviewers then provided comprehensive feedback, which was used to determine whether to accept the manuscript, reject it, or request revisions. The study findings show journal editorial process adherence to international editorial practices. These are plagiarism guidelines and blinded peer reviews. The participants indicated their acceptance of plagiarism guidelines. Asserting that plagiarism guidelines are usually applied suggests the journals’ commitment to this international practice using available tools, as captured in key informant interviews with a representative of the African-wide repository *(AW02_KII_KE)*: We usually do a plagiarism check using Turnitin. We have various ways in which we check that. Suggesting the seriousness of checking plagiarism, participants reported that journals are taking a step further by checking for AI plagiarism as well. This involves employing advanced software tools to scan manuscripts for similarities. A journal editor-in-chief *(JE10_KII_KE)* from Ethiopia reported that ‘These tools can quickly identify potential instances of plagiarism, helping editors maintain academic integrity and ensuring that authors’ work is original and properly cited’. Similar sediments were echoed by a journal editor-in-chief *(JE30_KII_KE)* from Nigeria, reporting ‘So, we have to make sure that we check for plagiarism; both normal plagiarism and AI plagiarism, so that when we publish our work, the papers, there shouldn’t be such issues.’

In addition, the online submission system was cited as part of internationally accepted peer review practices. The online submission system facilitates efficient management of peer reviews by assigning manuscripts to editors and reviewers. The system tracks the progress of articles and handles communication between editors, reviewers, and authors, thereby making the peer review process seamless.

*“We are strict with the process that they submit their articles directly from the online journal system or email the editor. Even when it is sent directly to the editor, it has to be put into an online journal system for processing”.* A Journal Editor-in-Chief from Kenya *(JE01_KII_KE).*

An internationally accepted, blinded peer review was stated to be a common practice in the African journal editorial *process.* A representative from an African-wide repository *(AW02_KII_KE)* reported, ‘Our peer review was double-blind. This means that the reviewers do not know the author, which is one of the reasons we insist on having a blinded manuscript.’ This promotes objectivity, as reviewers can assess the manuscript based on its quality without influence from authors’ affiliations or identities. Anonymity by blinded peer review safeguards the integrity of the review process and reduces conflicts of interest. However, in some instances, editorial practices and processes are not followed for several reasons, such as the economics of publishing. The business angle of publishing compromises editorial practice. A key informant *(IH01_KII_KE)* from an institutional research head in Kenya explained, ‘But for many African journals, you realize, especially those housed by either individual publishing houses or by universities, you will find that they are more of a business really.’

Like the selection process, financial concerns have been noted to affect adherence to editorial practices. It was also reported that cost [financial constraints] is a key reason for not following editorial practices. Financial constraints often hinder African journals from implementing effective editorial practices, as limited funding often restricts access to resources, such as peer reviewers and advanced submission and tracking systems. systems. A research director *(IH02_KII_KE)* from Kenya said, ‘There might be those reasons, financial reasons, and also if the editors of those journals are not exposed to so many reviewers.’

### Measures of transparency and rigor

Several measures of transparency and rigor in editorial practices, including accepting manuscripts, the peer review process, and production practices, were discussed as per journal’s focus. Participants detailed the process of accepting the manuscripts, including thorough pre-review checks. ‘The editor will have some pre-check then to invite the peer reviewers to do the review, and then, to make the decision whether the manuscript needed to be revised or accepted or rejected, out explained’ *(AW01_KII_KE, Africanwide repository_representative).*

In addition, an institutional repository *(IR15_KII_KE)* from Kenya alluded, ‘So, once a paper is received, the first step is to check whether the scope is within what the journal publishes, and once it is believed to be within the scope.’ Communication with authors and reviewers has also been reported as a measure of transparency and rigor. This communication convinces the author of the work that has gone through the review process. A journal editor-in-chief *(JE30_KII_KE)* from Kenya reported, We communicate that with the authors, ‘we inform them of their decision along with the constructive feedback that they need to know.’

The peer review decision-making process was alluded to by the study participants as critical for African journals and authors. Peer review is viewed by the authors as a measure of transparency and rigor. Participants indicated that decision making is based on reviewers’ comments, which is an aspect of transparency, as supported by the following quotes.

“ *When we get the review reports, we analyze them and send feedback to the authors who can go on and revise the paper according to the reviewers’ comments if the paper has been accepted for publication, and then after the revision, the other sends the paper back to us*” *(JE05_KII_KE, Journal editor-in-chief_Kenya)*

To enhance transparency, journal policy guidelines have been reported to be significant when making peer review decisions. These guidelines are essential, as they set clear standards for ethical conduct and reviewer responsibilities. Therefore, these guidelines help to maintain the integrity of the review process. For instance, a journal editor-in-chief *(JE12_KII_KE)* from Kenya said, ‘Generally, you may even find a journal not accepting the paper because the reviewers have not accepted the paper. Some reviewers even declined the papers because of a lack of understanding of the content. That is why you must be very precise about which reviewer you are sending your journal paper to, that’s the thing. After that happens, you make a decision based on the indicators that have been set by the journals that this is now matching our standards; we can now say yes, or we can say no. So that is entirely what happens.’

### Underlying factors affecting the editorial practices of African journals

**Challenges with peer review**

Consistency in the peer review process is a major factor that affects editorial practices. Reviewers may vary in their expertise, thoroughness, and evaluation standards. This inconsistency can lead to unequal assessments of manuscripts, which may affect the quality and fairness of the review process. A journal editor-in-chief *(JE30_KII_KE)* from Kenya reported that the consistency of the peer-review process is a major challenge in African journals, as explained in the following excerpt. ‘You know our journals struggle with maintaining consistency. So, it said several times you can start a journal and then we say that we shall publish maybe twice a year, and it does not happen.’

For an article to be accepted for publication, it must go through a thorough editorial process that includes peer review and editorial review. However, financial constraints are an underlying factor that affects editorial processes. Financial constraints limit activities, such as obtaining qualified editors and peer reviewers. Limited funding can also prevent journals from compensating their editors and reviewers for their time. This leads to a lack of motivation, which may compromise the peer review quality. Financial constraints were alluded to as a major problem with the peer review process by a journal editor-in-chief *(JE_FGD_NG)* from Nigeria. *‘*Maintaining the editorial office is one issue we had out of the funds we get from processing publication fees; we buy every infrastructure that is required within the editorial office. Next, we carry out plagiarism using Turnitin on all our manuscripts, which has to be paid for. We also translated all our abstracts into the French language, and again, we need to pay the editorial staff that does that. Therefore, all in all, every other editorial team member does it without requesting any salary or allowance from the team, both within the university in Africa and outside Africa. We do not pay anyone for editorial duties, but it is the only day before the running of the editorial office.’ A journal editor-in-chief *(JE_FGD_ET)* from Ethiopia added that ‘Pre-review contribution is a voluntary scientific contribution, but reviewers just want incentives, financial incentives.’

It was reported that technological infrastructure influences editorial workflow, such as improving the peer review process, integrating submission systems, improving editorial management, and reducing workload, as indicated in the following quotes.

“ *It makes the workflow very easy. For example, the installation of software such as OJS certainly makes the workflow seamless. The copy editor can, with just a click, assign manuscripts to different reviewers and send them back to the user.” (IR18_KII_KE, Librarian_Kenya).*

“ *In the last few years, we have had the AI revolution where you can be able to use technology to probably spend less time doing editorial work and maybe workflow scheduling. That is, if you can be able to have a technology that can be able to help to do such repetitive functions, that would be very helpful*” *(JE23_KII_KE, Journal editor-in-chief_Kenya*).

**Challenges in editorial board engagement**

The commitment of the editorial team is a key challenge in journal editorial practice, as a lack of dedication can lead to delays in peer review processes. Information from the study participants also highlights significant challenges for editorial board engagement. Key challenges include the commitment of editorial teams as elaborated by a journal editor-in chief *(JE03_KII_KE)* from Kenya, ‘some journals do not even have an editorial team; they do not even peer review; these are some of the issues that may impede African journals from being seen out there.’

Another challenge in editorial board engagement is obtaining editorial team members who are based outside Africa to meet the requirements of geographic representation; geographic representation on a journal’s editorial board ensures diverse perspectives and the ability to address regional research priorities effectively. This diversity helps address biases and steers a broader understanding of cultural and scientific contexts. The challenge of expert review was brought forward by an institutional research head *(IH06_KII_KE)* from Kenya. ‘When our journal wants to get into the international database, like the West, some may demand that the articles must be reviewed by experts who are not African themselves.’

Furthermore, journal editors-in-chief *(JE_FGD_NG)* from Nigeria highlighted challenges with committing time to review the manuscripts since they were also engaged in teaching within their respective universities. ‘One of the main challenges we are facing is that most of our journals are institutional-based. Therefore, the editorial team was also a part of the university. Therefore, they are not separated from the university’s running or the university system. Thus, we have a lot of work that is not committed to 100%, as it is being run in international journals. That is the only thing they do, and that is the number one thing that is one of the major challenges that we have. So, the rate at which we turn out publications might be affected a bit.’

**Scope of the journals**

The journal scope was reported to be a key factor for authors when selecting journals to publish their work. The scope determines whether the journal aligns with the author’s research area as mentioned by authors *(FGD_Authors_KE)* from Kenya ‘There are people who don’t go for a more general journal but would go for a specialized one. For example, if you are doing knowledge management, instead of going for a journal that is more general, you would rather go for a journal that addresses knowledge management.’ An author *(AU10_IDI_KE)* from Kenya also alluded to, ‘You are required by a professor, maybe to publish in a certain journal. So, for instance, for me, I was told I must publish in a journal called Mineral Engineering, which is one of the prestigious journals in our area.’

The study participants narrated niche journals are key when it comes to promoting specialized knowledge, dissemination of specific topics as well as promoting local knowledge as indicated by an author *(AU05_IDI_KE)* from Kenya, ‘There are niche journals which publish specific topics. Therefore, those journals have a very important space because it means minded individuals, researchers do put their works there.’

In addition, it has been reported that some authors will look for the target audience as well as the audience’s reach. Journals with well-defined audiences reach readers who will find their research most relevant and impactful very quickly, as explained by the author *(AU03_IDI_KE)* from Kenya, ‘I think it depends on the audience to whom you want to communicate. If you talk about even the members of the public, it’s about decision-makers in government agencies; it could be writing for NGOs; it could be writing for scientific communities such as researchers and academics.’

**Publication costs**

High fees can deter submissions, particularly in low-resource settings. Authors often opt for journals with affordable and waived fees, while maintaining high-quality publication standards. The article processing charges or publication costs were discussed as a major factor influencing journal selection, especially in the African research ecosystem by the author *(AU05_IDI_KE).* ‘One of the most common things researchers struggle with is the cost of publication. When a journal is charging two thousand or three thousand dollars for publication, it becomes unaffordable.’

**Journal credibility and trustworthiness**

The findings revealed that perceptions and opinions of academic and research quality and prestige influence African journals. For instance, the participants mentioned perceptions of credibility and trustworthiness. indicating that African journals are not good, and African research is poorly done, as explained in the following excerpts expressed in a focus discussion:

“ *You see, over the years, the perspective that everything is not coming from Europe from wherever it was considered so imperial. These derogatory things continue to affect some of us. Even if you come to people publishing in Asian journals and other journals thinking that the African journals are too imperial and historically based on colonial issues as you have mentioned because they used to be pioneers in terms of knowledge sharing that kind of thinking has been in the minds of many Africans to publish and other things”(JE_FGD_NG, Journal editor-in-chief_Nigeria).*

A journal editor in Kenya added:

“ *As I told you, the academic quality in Africa, to be frank, is poor. We don’t have a good quality of academics in Africa. So, already if the work is not up to the standard, can you imagine even at the editorial level, there is nothing much you can do because that is what you have to publish*” *(JE10_KII_KE, Journal editor-in-chief_Kenya).*

Journals that receive publications from high-profile authors are always perceived to be prestigious because these authors bring credibility and recognition to the journal. Their work attracts high citations and a wider readership, thus enhancing the journal’s academic reputation and influence.

“ *African journals are thought to be of lower quality or less prestigious and this can be attributed to the low status of authors publishing in them. Journals receiving publication from high profile authors tends to be perceived as having good quality” (JE18_KII_KE, Journal editor-in-chief_Kenya)*

Study participants alluded to the fact that journals with professional editorial teams are preferred more by authors because they are perceived to be credible, as indicated by a journal editor-in-chief *(JE02_KII_KE)* from Keya, ‘Composition of the editorial team or editorial board members, and then I’d say the professionalism accompanying the different editors. Therefore, if a journal has editors whose research ethics are questionable, then the journal would be questionable.

The participants further indicated that the geographic distribution of the editorial board was key for authors to gain trust in the journal. This distribution enhances the journal’s credibility and trustworthiness by attracting submission from a wider global audience. Authors *(AU_FGD_ET)* from Ethiopia alluded, ‘Diversity in editorial board is critical in terms of when you establish a journal that you need to have international big names as much as possible under your team to be part of the journal editorial board. Again, this depends on how much that specific university is doing as far as internationalization is concerned. Is it reaching out to global professors in this specific area? Is it partnering across various prestigious universities where they can access these kinds of pro professors?’.

It was also suggested that the history of a journal is critical when it comes to journal trustworthiness and credibility. The history of a journal reflects its consistency, reputation, and longevity in publishing high-quality works. Journals with proven track record are always viewed as more respectable and within the academic committee as alluded by an institutional repository representative *(IR17_KII_KE)* from Kenya ‘But you see now history now is the one that speaks some Journals which will assure you or copyright but once you publish with them even you are welcome. So, history of the journal.’

In addition, journal indexing has been reported to impact an author’s trust in a given journal. Indexing enhances the author’s trust by signifying that the journal meets the established international standards of quality, visibility, and credibility. The authors are more confident in submitting their articles to an indexed journal. Research disseminated in indexed journals is widely discoverable, as reported by an editor-in-chief *(JE12_KII_KE)* in Kenya, ‘Then another one is on the indexing and ranking. That is obviously clear that you will want to check whether the metric you want to look at the scores.’

Study participants stated that prestigious journals have a high impact factor, are usually open access, and contribute to a wider audience. These journals contribute to a wider audience by offering greater accessibility and visibility through major indexing databases and distribution networks. Their reputation attracts articles and therefore ensures the high impact of the published work and a broader dissemination as alluded to by an author *(AU09_IDI_KE)* from Kenya ‘A prestigious journal has a larger audience; you have a larger audience for your work. Therefore, it is very helpful to publish a work in a prestigious journal.’

## Discussion

In a field built upon coloniality, or the diffuse, systematic ways that knowledge derived from Western epistemic structures dominates and, through this, helps reinforce Euro-American global dominance ^25^; however, these platforms are a major medium through which epistemic injustice unfolds. ^1^ The results of this study highlight various editorial practices among African journals to enhance rigor and transparency in peer-review decision-making. African journals’ editorial practices are also affected by underlying factors, including financial constraints and challenges in technological and digital infrastructure. African journals also face the challenges of credibility and trustworthiness from the authors’ perspective.

This study revealed the variability in adherence to editorial practices among African journals. Editorial practices on receiving an article, such as copy editing, ethical review checks, proofreading, and artificial intelligence content checks, commence the entire process of editorial integrity. An earlier study showed that these processes before peer review are as important as peer review itself. ^26^

These findings imply that strengthening editorial practices at the pre-peer review stage is essential for enhancing the credibility and integrity of African journals. Without consistent adherence to processes like copy editing and ethical checks, journals risk compromising quality and trust, which may deter authors and limit their global competitiveness.

Journal peer review is an important process for improving journal credibility and trustworthiness among authors. The peer process includes blinded peer reviewing, plagiarism guidelines, reviewers assigned based on areas of expertise, collective decision-making, diverse editorial boards, clear editorial policies, track record keeping, and the global mapping of publications. Not following these editorial practices can be attributed to tedious, time-consuming, and costly processes. ^26, 27^ However, we established that some journals bypass the process because of time constraints, unavailability of reviewers, or financial challenges. These findings mirror previous studies that highlighted financial and logistical hurdles as barriers to consistent editorial standards. ^26^ A previous study established that financial constraints were the foremost challenge to editorial practices and could be attributed to neglect by donors ^27^ and the inability to be supported by the institutions that housed them. This implies that the journals are unable to pay remuneration for their editors.

Some African journals also face a major challenge in maintaining editorial integrity, which includes conflicts of interest, copyright issues, ghost authorship, inactive journal databases, commercialized publishing, and dual language publishing systems. However, in scholarly publications, identifying ghost authorship from an editorial perspective is quite challenging, despite it being an aspect of editorial integrity. ^28^ This reflects longstanding negative opinions about African research and journals and their supposed inferiority to other regions. ^29^ These factors led many African journals to have negative connotations because they lacked quality, integrity, and efficiency. Mirroring broader trends, while participants saw publishing in northern journals as colonial and limiting African journal visibility, most took their research to these journals before African ones because of the limiting factors of African journals, their negative perceptions of them, and the need to publish in high-impact journals for their work. These findings imply that unless African journals address issues undermining editorial integrity, they will continue to be perceived as lacking credibility and professionalism. This persistent perception fuels a cycle where African researchers prioritize publishing in northern journals, further marginalizing local journals and reinforcing epistemic inequalities in global knowledge production.

Other than peer review, factors that affect African journals’ credibility and trustworthiness include the journal’s impact factor, article processing charges (APC), journal scope, open access status, publication speed, audience reach, journal indexing, and acceptance rate. These findings are in line with earlier studies that established that the credibility and trustworthiness of the journal influence the author’s decision to publish in the journal. ^30, 31^ This study found that authors often prefer to publish in prestigious and niche journals, particularly those with high impact factors and broad visibility. This is because of their high impact factor, open access, and often contributing to a wider audience. Additionally, this study found that African journals tend to have a narrow scope, primarily involving African researchers. This is despite the existence of African journal online platforms that have increased the accessibility and visibility of African journals. ^32^ As much as we consider all these factors, having a professional editorial team remains a key factor for the authors. Therefore, journals need to address the issue of editors working without pay or support, and the overall challenges in maintaining editorial integrity, inconsistent publishing, and slow processes. Without addressing these structural and operational barriers, African journals will continue to struggle to attract high-quality submissions and global readership, limiting their role in shaping scholarly discourse and advancing locally relevant knowledge.

Inadequate funding of African journals results in underdeveloped technological and digital infrastructure, as they struggle with online visibility, managing submissions, and the basic logistics of running an academic publication. All of these struggles contribute to the invisibility of these journals on international platforms. The invisibility, therefore, prevents the invaluable knowledge in these journals from being disseminated widely because their impact factor remains low. Journals that garner high levels of impact and status are historically entrenched in northern journals. To gain the status and metrics needed to secure grants, earn promotions, and reap recognition, authors must publish in these high-impact journals. Because institutions often rely heavily on grant money in “soft money” financial systems, they often push their researchers to publish in high-impact journals. Epistemic injustice—the systematic and deep-rooted marginalization and erasure of knowledge and methods of knowing from formerly colonized regions—is baked into the global research system. ^1^ Journals are key conduits for the reproduction and naturalization of this system. These findings highlight how the systemic underfunding of African journals contributes to their marginalization in global scholarly discourse, reinforcing epistemic injustices that sideline African knowledge systems. Without equitable investment in local academic publishing infrastructure, the global research ecosystem will continue to privilege northern journals, limiting the visibility, credibility, and influence of research from the Global South.

The study had some limitations, which included a lack of buy-in from the respondents and failure by respondents to provide reliable information. The risks were mitigated by carrying out an inception meeting that enabled the participants to understand the aim of the study.

To improve the visibility of African journals, interventions should focus on strengthening editorial practices and fostering adherence to global standards while accommodating the unique context of African scholarship. Capacity-strengthening initiatives should leverage existing opportunities such as networking, collaborations, shared infrastructure and the use of open-access platforms to promote visibility and accessibility. A unified movement towards funneling resources into the development of African journals, a cultural shift for African researchers to publish in these journals instead of those in the North, and a push for African institutions to support their researchers publishing in continent-based journals should all be the bedrock of transformation. Therefore, more emphasis should be placed on editorial practices to solve the underlying factors of journal editorial practices.

## Conclusion

The visibility of African journals is critically influenced by the quality and consistency of their editorial practices. Addressing this pivotal factor requires targeted interventions that address underlying issues, such as limited awareness of visibility platforms, challenges in journal access and dissemination, financial constraints, and inadequate digital infrastructure. These challenges are deeply interconnected and affect the ability of African journals to adhere to high editorial standards and achieve global discoverability.

## Author contributions

• **Amboka P:** Conceptualization, data curation, formal analysis, investigation, methodology, project administration, resources, software, validation, visualization, and writing – Original Draft Preparation.
• **Krugman D**: Data Curation, Formal Analysis, Investigation, Methodology, Validation, Writing – Review & Editing.
• **Simiyu A**: Data Curation, Formal Analysis, Investigation, Methodology, Validation, Writing – Review & Editing
• **Kariuki H**: Data Curation, Investigation, Methodology, Writing – Review & Editing.
• **Ondiek B**: Data Curation, Investigation, Methodology, Validation, Writing – Review & Editing.
• **Orobaton N**: Funding Acquisition, Resources.
• **Igonya E**: Conceptualization, Data Curation, Formal Analysis, Investigation, Methodology, Resources, Software, Supervision, Validation, Writing, Review & Editing.
• **Neba A**: Funding Acquisition, Investigation, Methodology, Resources, Supervision, Writing, Reviewing, and Editing.
• **Vicente-Crespo M**: Conceptualization, Data Curation, Formal Analysis, Funding Acquisition, Investigation, Methodology, Resources, Supervision, Validation, Writing, Review & Editing.
• **Kirimi Sindi J**: Conceptualization, Data Curation, Formal Analysis, Funding Acquisition, Investigation, Methodology, Resources, Supervision, Validation, Writing, Review & Editing.

## Ethics and consent

The protocol and other study documents were approvedfirst approved by APHRC’s Institutional Review Board (IRB) and later by the Amref Ethics and Scientific Research Committee (ESRC), Kenya (Reference number: ESRC P1677/2024). The Amref ESRC approval was granted on June 18, 2024. Members of the study team underwent ethical training for researchers and received valid certifications for the study period.

Potential study participants provided a written informed consent to participate in the study with written and oral information about the study before any consent to participate was sought from the potential participants. They were asked to sign consent forms before the start of data collection. Specifically, they were adequately informed about the purpose of the study and the methods used. In addition, they were informed about the institutional affiliation of the researchers, anticipated benefits and potential risks and follow-up of the study, discomfort it may entail, the right to choose to participate or not participate in the study, or to withdraw consent to participate at any time, without any reprisal whatsoever. Measures were taken to ensure confidentiality of the information they provided. The respondents were asked to sign consent forms before the start of data collection. For online meetings, there was oral consenting, and participants signed consent forms before the start of data collection. Participation in this study was voluntary. The participants were informed of the length of the interview. For the physical interviews, participants were compensated for the transport they used to come to the interview venues.

## Data Availability

Associated data to this article have been published in Zenodo repository. Zenodo: Qualitative Dataset on the Editorial Practices of African Journals: A Case of Kenya, Ethiopia, Nigeria, and Mozambique [Data set]. Zenodo. https://doi.org/10.5281/zenodo.14602064. ^33^ Data are available under the terms of the Creative Commons Attribution 4.0 International license (CC-BY 4.0).
